# The origins and implications of glycerol ether lipids in China coastal wetland sediments

**DOI:** 10.1038/s41598-019-55104-y

**Published:** 2019-12-06

**Authors:** Xiaoxia Lü, Xiaolei Liu, Changgui Xu, Jinming Song, Xuegang Li, Huamao Yuan, Ning Li, Deying Wang, Hongming Yuan, Siyuan Ye

**Affiliations:** 1 College of Marine Science and Technology, China University of Geosciences, Wuhan, 430074 China; 2 Laboratory for Marine Geology, Qingdao National Laboratory for Marine Science and Technology, Qingdao, 266061 China; 3 Organic Geochemistry Group, MARUM Center for Marine Environmental Sciences, Bremen, 28334 Germany; 4 Tianjin Branch, CNOOC China Limited, Tianjin, 300459 China; 5 Institute of Oceanology, Chinese Academy of Sciences, Qingdao, 266071 China; 6 Bohai Oil Research Institute, CNOOC, Tianjin, 300459 China; 7 Key Laboratory of Coastal Wetland Biogeosciences, Qingdao Institute of Marine Geology, China Geological Survey, Qingdao, 266071 China

**Keywords:** Marine chemistry, Environmental chemistry

## Abstract

Coastal wetlands are terrestrial-marine transition zones harboring diverse active microbial communities. The origins of diverse glycerol ether lipids preserved in coastal wetlands are rarely investigated. 16 surface sediments were collected from the coastal wetland at Guangrao (GR), Changyi (CY) and Xiamen (XM), where both climate and sedimentary environment show significant differences. Ten groups of glycerol ether lipids, including isoprenoidal and branched glycerol dialkyl glycerol tetraethers (iGDGTs and bGDGTs), isoprenoidal and branched glycerol dialkanol diethers (iGDDs and bGDDs), hydroxylated isoprenoidal GDGTs and GDDs (OH-GDGTs and OH-GDDs), overly branched GDGTs (OB-GDGTs), sparsely branched GDGTs (SB-GDGTs), hybrid isoprenoid/branched GDGTs (IB-GDGTs) and a tentatively assigned H-shaped branched GDGTs (H-B-GDGTs) were detected and quantified. Sediments collected in the north (Guangrao and Changyi) contain, in general, a lower abundance of GDGT (3.7–55.9 ng/g sed) than samples from south (Xiamen; 251–1020 ng/g sed). iGDGTs and bGDGTs are the predominant components at all sites and account for 17.2–74.3% and 16.1–75.1% of total ether lipids, respectively. The relative abundance of iGDGTs decreases but that of bGDGTs increases with the distance from sea, suggesting a marine vs. terrestrial origin of iGDGT and bGDGTs, respectively. In addition, the methylation index (MI_OB/B/SB_) of branched GDGTs shows a significant inverse correlation with water content, suggesting that marine waters have a major influence on the microbial communities inhabiting wetland sediment. Such an assumption was confirmed by the distinct lipid pattern of three low water content (<5%) samples collected in an area isolated from tidal flushing. The other isoprenoidal ether lipids, such as iGDDs, OH-GDGTs and OH-GDGTs, have a similar distribution as iGDGTs, indicating a common biological source, so do the corresponding non-isoprenoidal ether lipid series with bGDGTs. The BIT value increases with increasing distance from the sea, which implies that the BIT index can be probably applied to trace past sea level change in costal wetland settings. The reconstructed temperature from TEX_86_ shows significant offset from observed data, but only little deviation for the MBT/CBT calculated temperature. This suggests that the MBT/CBT has the potential to reconstruct past temperatures in coastal wetland settings.

## Introduction

Coastal wetlands are terrestrial-marine transitional zones, harboring highly abundant and diverse biota communities, which are very sensitive to environment and climate change. The environment and climate change can cause the vegetation and biota communities changing correspondingly^1,2^.

**Figure 1:**
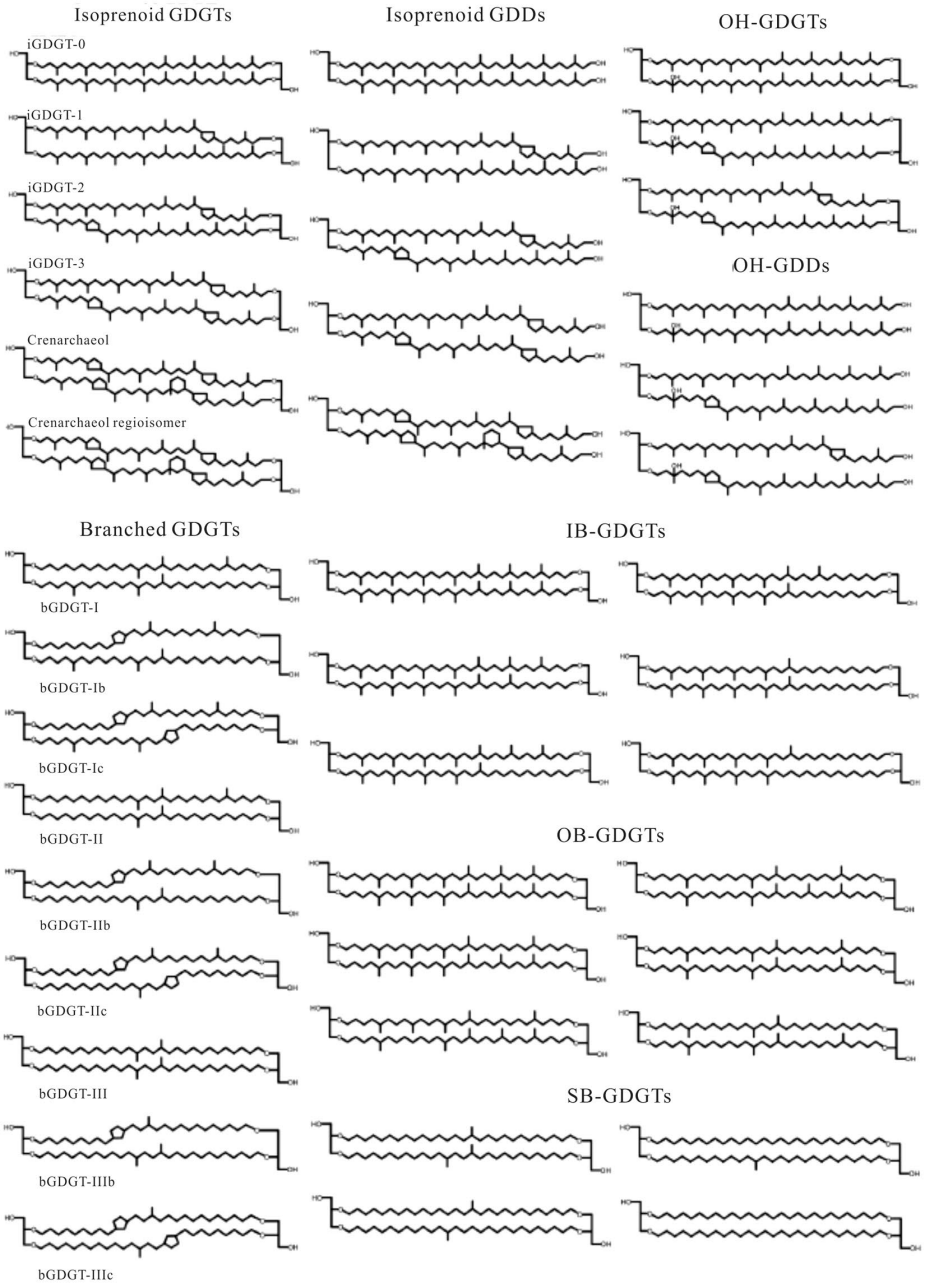
The molecular structures of glycerol ether lipids.

Microorganisms are the primary agents of geochemical change, and their lipids can be well preserved in wetland. Glycerol dialkyl glycerol tetraethers (GDGTs) are membrane-crossing bipolar lipids commonly synthesized by most Archaea and a few bacterial species. GDGTs found in various geological environments are of two main types: the archaeal isoprenoidal glycerol dialkyl glycerol tetraethers (iGDGTs) and the non-isoprenoidal branched glycerol dialkyl glycerol tetraethers (bGDGTs), which are likely produced by soil bacteria^3,4^ (Fig. 1). Recently, novel glycerol ether lipids, including hydroxylated isoprenoidal GDGTs (OH-GDGTs), hybrid isoprenoidal/branched GDGTs (IB-GDGTs), hydroxylated isoprenoidal GDDs (OH-GDDs), overly branched GDGTs (OB-GDGTs), sparsely branched GDGTs (SB-GDGTs) and H-shaped branched GDGT (H-B-GDGT, such as H-1020) had been detected in various marine sediments^5–7^ (Fig. 1). The predominant distribution of IB-GDGTs, OB-GDGTs, SB-GDGTs and H-B-GDGT in the oxygen minimum zone (OMZ) and the deep anoxic water column suggested them *in situ* production in anoxic environment^8,9^.

GDGT based proxies, such as TEX_86_ (Tetra Ether indeX of tetraethers, consisting of 86 carbon atoms), MBT/CBT (MBT: the methylation index of branched tetraethers; CBT: the cyclization ratio of branched tetraethers), were established to trace the sea surface temperature (SST), mean annual air temperature (MAAT) and soil pH, respectively^10,11^. These molecular proxies have been applied in many studies for reconstructing the past climate or environmental change^12–15^. BIT (Branched and Isoprenoid Tetraether index) is a proxy to estimate the relative abundance of terrestrial versus marine organic matter in sediments^16^. A new methylation index (MI_OB/b/SB_), defined by the degree of methylation of OB-, B- and SB-GDGTs was proposed to trace the anoxic environment^8^.

China has the fourth longest coastlines in the world – around 18,000 km, presenting a large variety of ecosystems from the tropical to sub-tropical to temperate realms. The coastal ecosystems include mangroves, coral reefs, beaches, cliffs and salt marshes, and correspondingly, the coastal wetland in different regions is different. How microbial communities adapt to the coastal wetland ecosystems and whether the GDGTs-related proxies can be applied for environmental study is unknown. The correlation between microorganisms and wetland environment change and their interactions have not been well studied. In this work we investigated the lipids distribution in China coastal wetland to study the impact of various environmental factors on glycerol ether lipid producing microorganisms, and further testified the applicability of GDGT based proxies.

## Material and Methods

### Sampling sites

**Figure 2:**
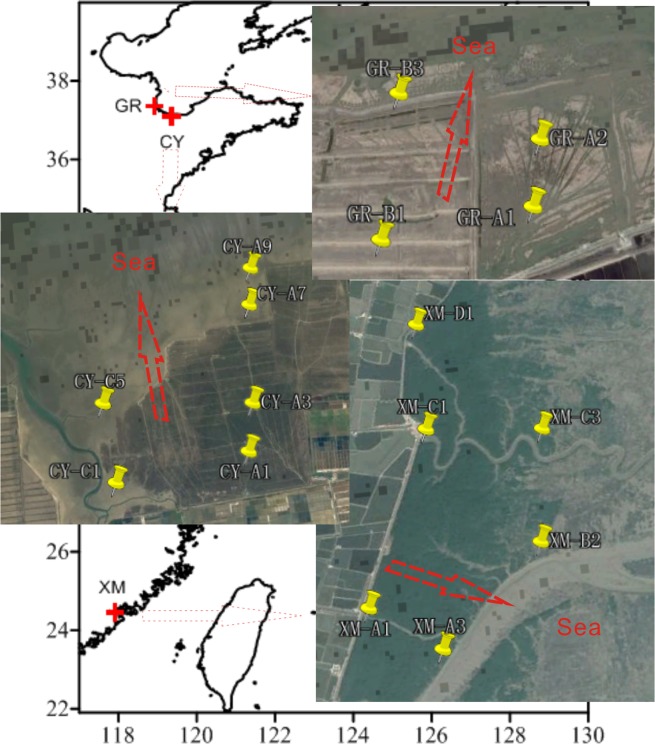
Sampling sites in China coastal wetland (CY: Changyi; GR: Guangrao; XM: Xiamen) (Generated by the software of Coreldraw X4. The satellite imagery was downloaded from http://earth.google.com).

We collected 16 surface sediments (at a depth from 0 to 2 cm) from China coastal wetland in different regions (Fig. 2), including 6 from Changyi (CY) and 4 from Guangrao (GR)) collected in June, 2012, and 6 samples from Xiamen (XM) in September, 2012. All samples were stored in a refrigerator at −20 °C until analyzed.

CY and GR wetlands locate at the Shandong province in northeastern China, where the climate (e.g. temperature and precipitation) is mainly controlled by Siberian-Mongolia High Winter Monsoon, accompanying with some influence of East Asian Summer Monsoon^17,18^. Therefore, the climate is cool and dry, and the mean annual air temperature (MAAT) is about 12.6 °C, mean annual precipitation (MAP) ~568.5 mm and the mean annual evaporation (MAE) ~1754.5 mm (http://cdc.cma.gov.cn/). However, the micro-environment in these two regions shows a little difference. For example, the pH in GR wetland is higher than that in CY (Table 1). The vegetation is also different. CY is dominated by *suaeda* and *tamarix*, but in GR is all *suaeda*. In addition, drier sediments in CY-A7, CY-A3 and CY-A1 are caused by a dam between site CY-A9 and CY-A7 which isolates them from high tide water (Table 1). XM wetland is in the Fujian province, south China where the MAT, MAP and MAE are about 20.4 °C, 1349.0 mm and 242.9 mm, respectively (http://cdc.cma.gov.cn/). The warm and humid climate in XM wetland is distinctive of that in CY and GR. The vegetation in XM wetland is dominated by mangrove.

**Table 1:** The concentrations of glycerol ether lipids and the corresponding environmental factors in China coastal wetland.

No.	Sites	Longitude (°E)	Latitude (°N)	pH	Water content (%)	TOC (%)	bGDGTs	iGDGTs	bGDDs	iGDDs	H-B-GDGT	IB-GDGTs	OB-GDGTs	SB-GDGTs	OH-GDGTs	OH-GDDs
							ng/g									
1	CY-A1	119.366	37.081	8.1	0.49	0.81	28.1	5.9	2.4	0.8	0.20	0.03	0.10	0.06	0.08	0.03
2	CY-A3	119.368	37.090	8.2	0.48	0.45	34.0	21.1	2.3	2.0	0.02	0.02	0.08	0.04	0.11	0.02
3	CY-A7	119.370	37.109	7.6	3.91	0.71	21.5	26.9	1.1	1.0	0.07	0.21	0.38	0.15	0.22	0.02
4	CY-A9	119.372	37.116	7.8	21.58	0.12	0.8	2.8	0.1	0.2	0.02	0.04	0.07	0.05	0.11	0.02
5	CY-C1	119.334	37.078	8.5	12.65	0.41	7.2	5.7	0.4	0.4	0.13	0.40	0.68	0.33	0.14	0.01
6	CY-C5	119.333	37.093	7.9	21.78	0.15	1.0	4.0	0.1	0.1	0.02	0.05	0.08	0.05	0.03	0.00
7	GR-A1	118.932	37.343	9.0	10.29	0.19	10.5	23.6	0.4	1.3	0.11	0.43	0.86	0.31	1.91	0.07
8	GR-A2	118.932	37.345	9.4	5.42	0.20	15.6	16.7	0.6	1.3	0.17	0.86	1.57	0.46	0.98	0.06
9	GR-B1	118.926	37.342	9.1	8.20	0.23	4.7	5.8	0.2	0.5	0.08	0.19	0.36	0.16	0.38	0.04
10	GR-B3	118.927	37.347	9.0	26.38	0.24	15.8	12.8	0.5	0.8	0.21	0.96	2.12	0.42	0.69	0.04
11	XM-A1	21.349	117.906	7.5	49.18	3.81	769.6	243.0	23.6	17.0	6.82	18.23	44.99	10.26	8.83	0.42
12	XM-A3	31.506	117.909	7.5	43.85	1.55	245.7	202.7	9.6	16.6	3.66	10.85	15.02	3.92	9.47	0.42
13	XM-B2	47.414	117.913	8.4	56.15	1.24	107.3	141.0	5.5	13.8	2.34	4.39	8.01	2.10	7.50	0.33
14	XM-C1	32.251	117.909	7.5	47.95	2.38	304.8	203.4	15.4	17.6	4.08	11.81	21.41	7.18	8.38	0.40
15	XM-C3	49.539	117.914	7.7	41.42	1.62	280.2	325.8	12.0	28.0	5.46	11.98	19.82	5.68	15.84	0.77
16	XM-D1	32.337	117.909	8.4	39.80	2.22	292.5	139.7	9.1	9.9	3.50	7.59	23.49	4.67	5.29	0.27

### Experimental procedure

All the samples were dried by a freeze dryer before extraction and homogenized with a pestle and mortar. 16 samples from China coastal wetland were extracted using a modified Bligh and Dyer protocol as described by Sturt *et al*.^19^ in MARUM Center for Marine Environmental Sciences, Bremen, Germany. The total lipid extract (TLE) was stored at −20 °C.

An aliquot of TLE of each sample was dissolved in 100 µL *n*-hexane/propan-2-ol (99.5:0.5, v:v) for HPLC-MS analysis. The lipids were analyzed by a Dionex Ultimate 3000 Ultra-High Performance Liquid Chromatography (UHPLC) coupled to a Bruker maXis ultra-high resolution quadrupole time-of-flight mass spectrometer (qTOF-MS), equipped with an APCI II source. The method was described in detail by Becker *et al*.^20^. Compound separation was achieved using two coupled Acquity BEH Amide columns (2.1 × 150 mm, 1.7 µm; Waters, Eschborn, Germany) maintained at 50 °C. GDGTs were eluted using the following gradient with eluent A [*n*-hexane] and eluent B [*n*-hexane: isopropanol (90:10, v:v)] at 0.5 ml min^−1^: the initial gradient was 3% B to 5% B in 2 min, followed by increasing B 10% Bin 8 min, to 20% in 10 min, to 50% in 15 min and 100% in 10 min. The column was washed with 100% B for 6 min at 0.6 ml min^−1^. Finally, the column was equilibrated with 3% B for 9 min. Detection of glycerol ether lipids was achieved using positive ion APCI, while scanning a *m/z* range from 150 to 2000. Each compound was detected using the corresponding [M + H]^+^ in the extracted ion chromatogram (Liu *et al*., 2012a, b, c). The concentration of each compound was calculated based on the peak area integration of [M + H]^+^ versus the peak area of C46 GTGT standard. The final concentration can only be considered semi-quantitative because we did not determine the response factors for GDGTs vs. the C_46_ GTGT standard.

## Results

### The total glycerol ether lipids composition in wetland sediments

**Figure 3:**
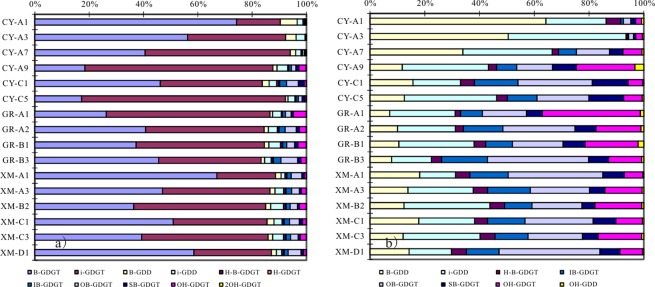
All detected glycerol ether lipids distribution (**a**) and the glycerol ether lipids without iGDGTs and bGDGTs (**b**) in China coastal wetland sediments.

Ten groups glycerol ether lipids were detected in China coastal wetland sediments, including isoprenoidal GDGTs (iGDGTs), isoprenoidal glycerol dialkanol diethers (iGDDs), hydroxylated isoprenoidal GDGTs and GDDs (OH-GDGTs and OH-GDDs); branched GDGTs (bGDGTs), branched GDDs (bGDDs), overly branched GDGTs (OB-GDGTs), sparsely branched GDGTs (SB-GDGTs), hybrid isoprenoid/branched GDGTs (IB-GDGTs) and a tentatively assigned H-shaped branched GDGTs (H-B-GDGTs). The glycerol ether lipids concentration in CY and GR sediments is in the range of 4.1–60.5 ng/g dry sediment, which is more than one magnitude lower than XM sediments, 294.6–1147.4 ng/g. Among all the glycerol ether lipids, iGDGTs and bGDGTs are the dominant component, count together for more than 80% of total ether lipids, in all analyzed sediments (Fig. 3a).

### Lipid distribution in each glycerol ether lipid group

**Figure 4:**
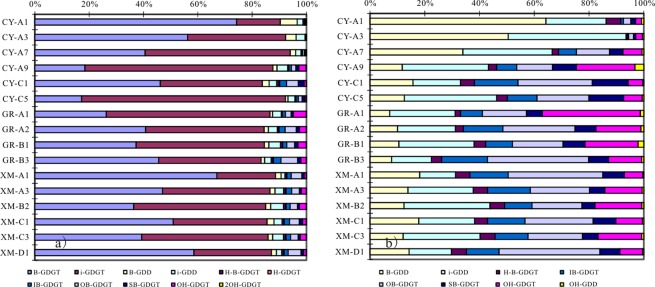
The distributional plot of predominant glycerol ether lipids (**a**) isoprenoidal GDGTs; (**b**) branched GDGTs).

In general, the lipids in the sediments collected from the three drier sites (CY-A7, CY-A3 and CY-A1) where the water content (WC) is <5% exhibit a very distinct distribution pattern from those in the other sites (Fig. 3b, Table 1). The GDGTs distribution shows a pattern that the relative abundance of bGDGTs increases with the distance increasing from sea, on the contrary, the relative abundance of iGDGTs decreased (Fig. 3a,b). The iGDGTs distributions in the three studied sites show different pattern. The most obvious difference is that the relative abundance of iGDGT-1, 2, 3 and crenarchaeol regioisomer (cren. iso.) is much higher in the three samples with WC < 5% in CY wetland. The relative abundance of cren. iso. is found higher in north than in south (Fig. 4a).

The branched GDGT distribution pattern can be divided into three groups. Group I refers to the three sites with water content <5% in CY wetland, Group II refers to the sites except the lower water content sites in the north wetland and Group III refers to the sites in the south wetland (XM). Group I is characterized with the highest abundance of bGDGT-II. Group II is characterized with the higher abundance of bGDGT-I, II and III. Group III is characterized with the highest abundance of bGDGT-I (Fig. 4b).

## Discussion

### Glycerol ether lipids origin

iGDGTs are membrane lipids of Group I.1 thaumarchaeota (including group I.1a and I.1b) and euryarchaeota^21–23^, and the bGDGTs are likely produced by anaerobic bacteria thriving predominantly in soil and peat^4,24^. Later studies also revealed unknown microbial sources in specific marine and lacustrine settings^25–27^. The distribution of iGDGTs versus bGDGTs (Fig. 3a) indicates that the iGDGTs are mainly contributed by marine thaumarchaeota and bGDGTs by terrestrial bacteria in China coastal wetland.

The iGDGTs distribution is characterized with lower relative abundance of cren. iso. in south wetland, which is different from the iGDGTs distribution in sea surface sediments that the relative abundance of iGDGT-1, 2, 3 and cren. iso. increased with the decreasing latitude^28^. This phenomenon suggests that the iGDGTs in coastal wetland have either different sources or different correlation to certain environmental factors. As we known, the relative abundance of iGDGT-1, 2, 3 and cren. iso. is higher in soil Thaumarchaeota group^29^. The high relative abundance of iGDGT-1, 2, 3 and cren. iso. in the three sites with WC < 5% may be contributed to the soil thaumarchaeota group (including group I.1a and I.1b) blooming, which suggests the micro-organisms could change their group to adapt to the environment change. The higher abundance of cren. iso. in most samples from north wetland is also due to the low marine thaumarchaeota. The result suggests that the arid environment caused by higher evaporation in north can cause the micro-organisms change.

Branched-GDGTs are thought mainly synthesized by members of the domain of bacteria, although it is still unclear which group does the source organism belong to^24,30^. The significant difference of bGDGTs distribution in drier and wetter sediments (Fig. 4b) suggests that the bGDGTs in the two types of samples are from different species. The different bGDGTs distribution between south and north wetter surface sediment may be due to the following causes: 1) the premier organisms of bGDGTs in south wetland are different from those in north; 2) the organisms adjust the bGDGTs compositions to adapt to the environment change from south to north. Further works are needed to investigate which is the controlling factor to result in the different distribution.

**Figure 5:**
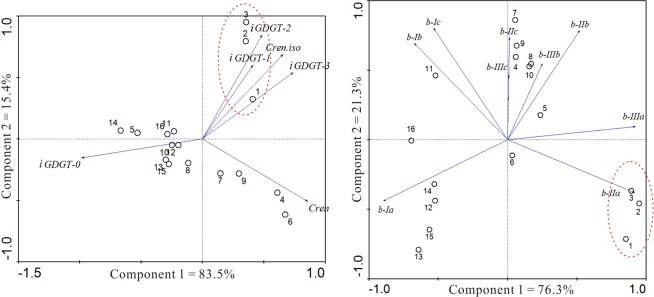
The principal components analysis (PCA) plots of the isoprenoidal and branched GDGT distribution in China coastal wetland. The circles and blue lines represent scores and response variables. Numbers in the plots correspond to the sites in Table 1.

Principal component analysis (PCA) was performed for the lipids distribution in the samples analyzed. Obviously, the three samples with WC lower than 5% show significant difference with other ones based on whether iGDGTs or bGDGTs distributions (Fig. 5). The result suggests that the water losing from sediment compels the organisms’ community change, or that the organisms adjust their membrane lipid compositions to adapt to the environment change.

Isoprenoid and branched glycerol dialkanol diethers (iGDD and bGDD) were interpreted as potential biosynthetic intermediates and/or degradation products of the corresponding GDGTs^5,31^. OH-GDGTs in sediments were thought mainly originate from the planktonic archaea^7,32,33^ due to the low activity of Thaumarchaeota in marine sediments^34^. OB- and SB-GDGT are from unidentified planktonic microorganisms favoring anoxic marine environment^8,9^. The source of IB-GDGT and H-B-GDGT remains elusive.

In China coastal wetland (whether in north or south), the concentrations of iGDD and bGDD show significant correlation with their corresponding tetraethers, iGDGT and bGDGT (Table 2). Our results are consistent with the hypothesis that the iGDD and bGDD may be the biodegradation production and/or share the same biological source with the corresponding GDGTs^5,31^. The correlation between other minor glycerol ether lipids and predominant glycerol ether lipids in north wetland is different from that in south. In north wetland, the minor glycerol ether lipids, including H-B-GDGT, IB-GDGT, OB-GDGT, SB-GDGT, OH-GDGT and OH-GDD have no significant correlation with iGDGT and bGDGT (Table 2). However, in the wetland sediments except the three drier sites (water content (WC) < 5%), H-B-GDGT, IB-GDGTs, OB-GDGTs and SB-GDGTs show a significant correlation with bGDGTs, while OH-GDGT and OH-GDD show a significant correlation with iGDGTs. The result may have the implications that: 1) H-B-GDGT, IB-GDGT, OB-GDGT and SB-GDGT have highly related biological source with bGDGT, and the same for OH-GDGT and OH-GDD with iGDGT in the wetter north samples; 2) the microbial communities in lower water content (WC < 5%) samples show significant difference from those in the wetter samples in the same region. In south wetland, the OH-GDGT and OH-GDD show significant correlation with iGDGTs and H-B-GDGT, IB-GDGT, OB-GDGT and SB-GDGT show significant correlation with bGDGT, which also suggests that the OH-GDGT and OH-GDD have the similar biological origin with iGDGT and the other minor branched glycerol ether lipids have the similar biological origin with bGDGT. Obviously, the relative abundances of minor glycerol ether lipids, especially IB-GDGT, OB-GDGT, SB-GDGT and OH-GDGT are higher in the wetter samples than in the drier ones, which suggests that the organisms related with these glycerol ether lipids are habited in the wetter environment. The result is consistent with the findings that the OB-GDGT and SB-GDGT are likely produced by hobbies in anoxic environment^8,9^ and that the OH-GDGT are of aquatic origin in marine environment^7,32,33^. However, sedimentary production of these minor glycerol ether lipids by related species dwelling within sediment and terrestrial environment cannot be excluded.

**Table 2:** The correlation between different glycerol ether lipids in China coastal wetland sediments.

Wetland	Number			bGDD	iGDD	H-B-GDGT	IB-GDGT	OB-GDGT	SB-GDGT	OH-GDGT	OH-GDD
North wetland	10	bGDGT	R	0.938	0.805	0.283	−0.036	−0.003	−0.099	−0.079	0.144
			p	0.000	0.005	0.428	0.921	0.994	0.786	0.829	0.692
		iGDGT	R	0.306	0.796	0.031	0.241	0.242	0.260	0.507	0.446
			p	0.391	0.006	0.933	0.502	0.500	0.468	0.135	0.196
	7	bGDGT	R	0.947	0.851	0.955	0.981	0.964	0.966	0.611	0.734
			p	0.001	0.015	0.001	0.000	0.000	0.000	0.145	0.061
		iGDGT	R	0.706	0.953	0.563	0.614	0.600	0.689	0.976	0.884
			p	0.076	0.001	0.188	0.142	0.154	0.087	0.000	0.008
South wetland	6	bGDGT	R	0.947	0.069	0.879	0.897	0.985	0.926	0.008	0.054
			p	0.004	0.897	0.021	0.015	0.000	0.008	0.988	0.919
		iGDGT	R	0.470	0.949	0.750	0.641	0.327	0.477	0.931	0.950
			p	0.346	0.004	0.086	0.170	0.527	0.338	0.007	0.004

### The implication of glycerol ether lipids distribution

#### The implication of GDGT-based proxies

**Figure 6:**
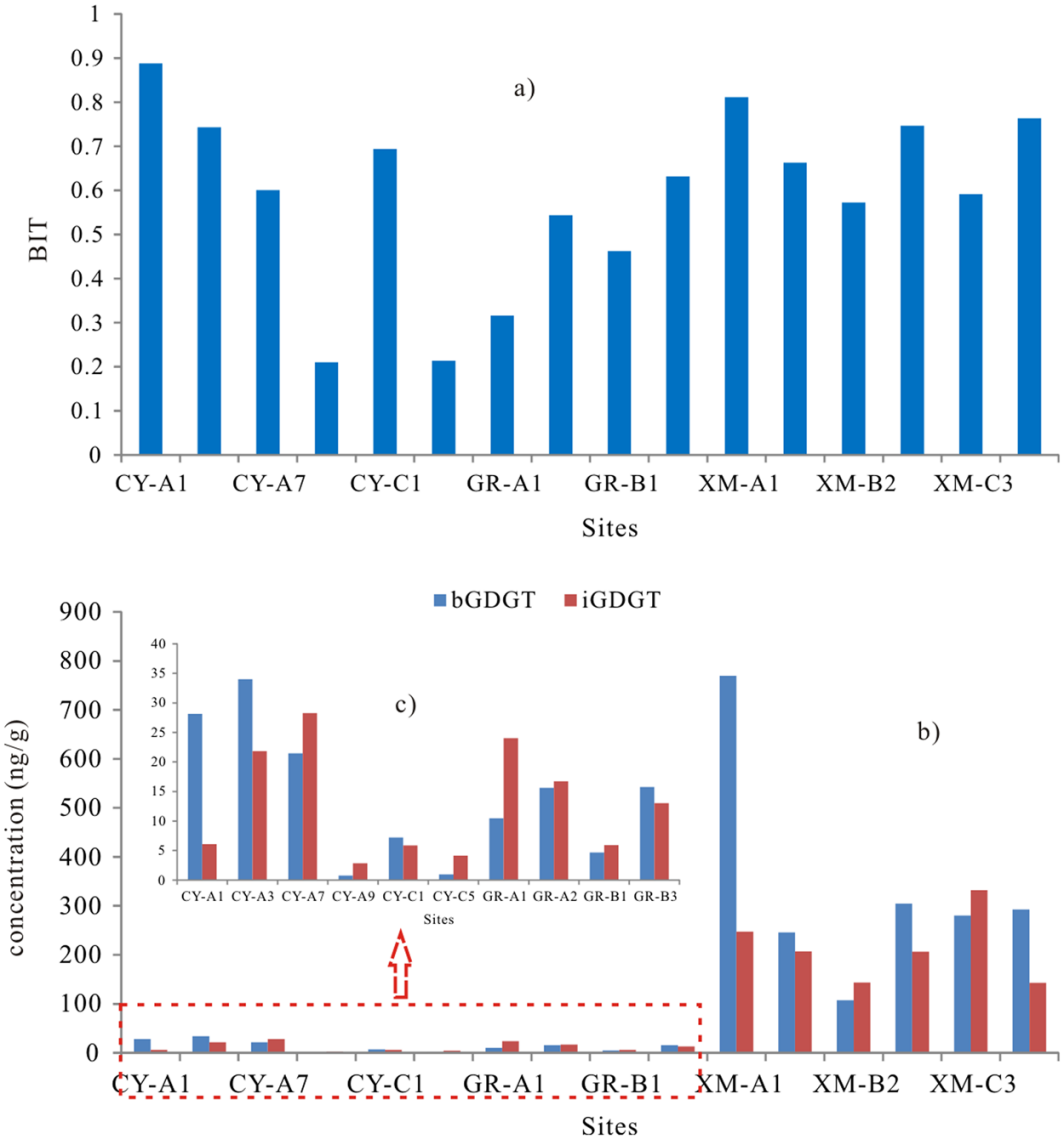
The BIT (**a**) and the concentration of iGDGTs and bGDGTs distribution (**b,c**) in China coastal wetland.

The BIT is used as a means to quantify the relative abundance of terrestrial organic matters (OM) in marine and lacustrine environment^16,35–37^. The BIT values are in the range of 0.2–0.9, which suggests the higher relative contribution of terrestrial OM. Obviously, the BIT value increases with the increasing distance from sea whether in north wetland or in south wetland (Figs. 6 and 2). The result also suggests that the iGDGT mainly come from the marine thaumarchaeota group I.1a though the soil thaumarchaeota group I.1b cannot be excluded (section 4.1). In GR wetland, we found the BIT values are higher in the samples near to the river (GR-A2 and GR-B3) than the others far away from the river (GR-A1 and GR-B1) though the concentration of iGDGTs in GR-B3 becomes higher (Figs. 6, 2 and Table 1). The result indicates that the bacteria take more advantage of the fresh water environment than archaeota. Furthermore, we found the BIT values change more significantly in north wetland than in south wetland, which may be due to strong evaporation resulting in the environment varying and further causing the communities of organisms changing with the increasing distance from sea in north wetland. Therefore, the BIT value in coastal wetland can refer to the relative distance from sea, especially in the wetland with high evaporation. Based on this, the BIT value can be as an indicator to trace the sea level change in the geological history.

**Figure 7:**
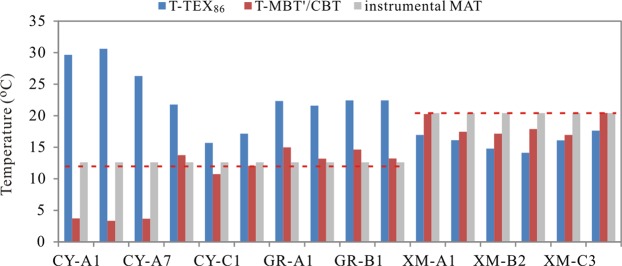
The reconstructed temperature from MBT/CBT and TEX_86_ (The red dot line represents the instrumental MAT in north and south wetland respectively).

The application of TEX_86_ in coastal environment is constrained for the terrestrial input and the BIT index value is used to assess the bias of the reconstructed SST^28,38,39^. The BIT values in the research samples are higher than 0.2, which indicates that the TEX_86_ may not be applied in coastal wetland. Actually, the reconstructed temperature from TEX_86_ shows a large bias to the instrumental MAT, and the residual temperature is from −6.3 °C to 17.0 °C, and the bias in the north appears higher than that in the south wetland, especially in the three drier samples (Fig. 7). The result also suggests that the index obtained from the marine environment (TEX_86_) cannot be applicable in the wetland, especially in the north wetland.

The bGDGT in the three wetlands are the terrestrial bacteria production, and thus their distributions should be influenced by the surrounding environment. The proxies of MBT and CBT, as the signals of bGDGT distribution should be recorded the information of environment change. Obviously, the reconstructed temperature from MBT′/CBT^40^ is close to the instrumental MAT except the three samples with WC < 5% in CY wetland (Fig. 7), and the residual temperature is from −3.5 °C to 2.4 °C, which is in lower than the RMSE (5 °C) obtained by Peterse *et al*.^40^. The result suggests that the MBT′/CBT can be used to trace the MAT in coastal wetland except the sites where the environment was disturbed by human activities.

More obviously, in the samples with water content lower than 5%, the temperature whether from TEX_86_ or from MBT′/CBT shows a much larger bias to the instrumental temperature, which suggests that the water loss due to the man-made dam may cause the microbial community change or the microorganisms adjust their lipids structure to adapt to the environment change. The result further suggests the interaction between environment and microorganisms.

#### The implication of minor glycerol ether lipids based proxy

**Figure 8:**
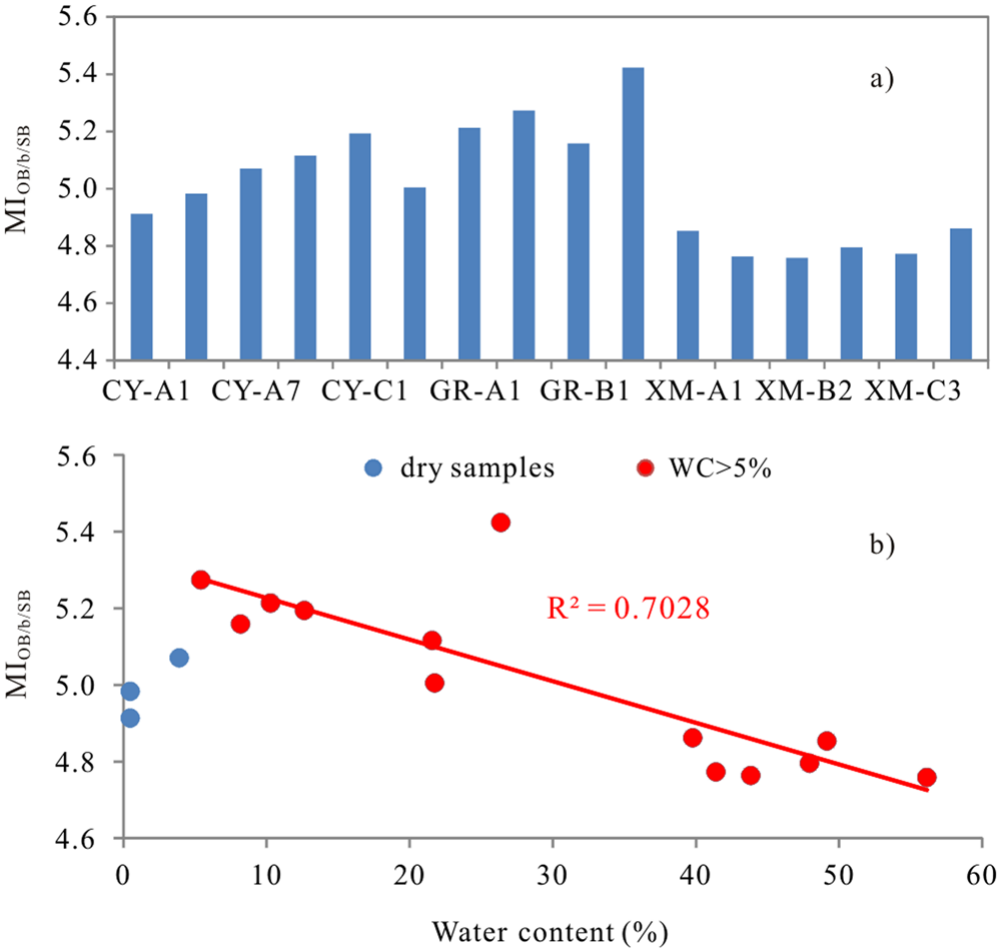
The MI_OB/b/SB_ distribution (**a**) and its correlation with water content (%) (**b**) in China coastal wetland.

The methylation index (MI_OB/b/SB_) of OB-GDGT, bGDGT and SB-GDGT (Eq. 1) was found to be as a gauge of redox conditions in marine environment, and the higher MI_OB/b/SB_ value refers to the more anoxic condition^8^. The MI_OB/b/SB_ values are from 4.8 to 5.4, which are in the range of 3.4–8.5 obtained from Black Sea and Cariaco Basin^8^. However, the MI_OB/b/SB_ values in XM mangrove sediment are lower than those in CY and GR wetland (Fig. 8a). As we know, the TOC and water contents in XM wetland are much higher than those in CY and GR wetland (Table 1), that means that the XM wetland is in more anoxic condition than CY and GR wetlands. This suggests that the MI_OB/b/SB_ in wetland cannot be used to trace the redox condition as well as in marine environment. Furthermore, we found that the MI_OB/b/SB_ shows a negative linear correlation with water content (p < 0.05) if the three dry samples excluded (Fig. 8b). The result further suggests that marine waters have a major influence on the microbial communities inhabiting wetland sediment, and more work needs to be done to clarify the origins of OB-GDGT and SB-GDGT and their response to environment change.

1 $$\begin{array}{rcl}{{\rm{MI}}}_{{\rm{OB}}/{\rm{b}}/{\rm{SB}}} & = & ([{{\rm{SB}}}_{980}]+2[{{\rm{SB}}}_{994}]+3[{{\rm{SB}}}_{1008}]+4[{{\rm{b}}}_{1022}]\\  &  & +\,5[{{\rm{b}}}_{1036}]+6[{{\rm{b}}}_{1050}]+7[{{\rm{OB}}}_{1064}]+8[{{\rm{OB}}}_{1092}]+10[{{\rm{OB}}}_{1106}]\\  &  & +\,11[{{\rm{OB}}}_{1120}]+12[{{\rm{OB}}}_{1134}])/([\sum {\rm{SB}}]+[\sum {\rm{b}}]+[\sum {\rm{OB}}])\end{array}$$

## Conclusion

Ten groups of glycerol ether lipids, including iGDGT, bGDGT, iGDD, OH-GDGT, OH-GDD, bGDD, OB-GDGT, SB-GDGT, IB-GDGT and H-B-GDGT were detected in China coastal wetland. The concentration of ether lipids in mangrove wetland (XM) is much higher than that in sandy-mud wetland (CY and GR). Among all ether lipids, the iGDGT and bGDGT are all the predominant compositions, and they account for 17.2–74.3% and 16.1–75.1% of total ether lipids.

The source of ether lipids are not only controlled by climate but also influenced by the micro-environment. In the three wetlands, the relative abundance of iGDGTs decreases with the distance increasing from sea, while that of bGDGT increases, which suggests that the iGDGT mainly originate from marine environment and bGDGT mainly originate from terrestrial organisms. However, the microbial communities in the three regions show a little difference. In XM wetland, the iGDGT mainly originate from marine thaumarchaeota group I.1a, while in CY and GR wetlands, the iGDGT originate not only from marine thaumarchaeota group I.1a but also from soil thaumarchaeota group, especially in the three dry sites (WC < 5%). The significant different distribution of bGDGT in dry and wet sediments suggests that the bGDGT in the two type samples may come from different species, which further suggests that the water content losing in the same region can compel the microbial communities change correspondingly. Among the minor glycerol ether lipids, the iGDD, OH-GDGT and OH-GDGT appear to have the similar biological origin with iGDGT, and IB-GDGT, OB-GDGT, SB-GDGT and bGDD show the similar origin with bGDGT in the samples with WC > 5%. In addition, the methylation index (MI_OB/B/SB_) of branched GDGTs shows a significant inverse correlation with water content, suggesting that marine waters have a major influence on the microbial communities inhabiting wetland sediment.

The BIT value increases with the increasing distance from sea, which means that the BIT value can be used to trace the relative distance from sea, and which can further be used to trace the sea level change, especially in the area with high evaporation. The TEX_86_ could not be applicable to trace the temperature change due to the high soil organisms’ input. On the contrary, the MBT/CBT has the potential to trace the paleotemperature if the wetland has not been disturbed by human activities.
